# Augmentation of Dispersion Entropy for Handling Missing and Outlier Samples in Physiological Signal Monitoring

**DOI:** 10.3390/e22030319

**Published:** 2020-03-11

**Authors:** Evangelos Kafantaris, Ian Piper, Tsz-Yan Milly Lo, Javier Escudero

**Affiliations:** 1 School of Engineering, Institute for Digital Communications, University of Edinburgh, Edinburgh EH9 3FB, UK; javier.escudero@ed.ac.uk; 2 MRC Centre for Reproductive Health, Department of Child Life and Health, University of Edinburgh, Edinburgh EH9 1UW, UK; ian.piper@brainit.org; 3 Royal Hospital for Sick Children, NHS Lothian, Edinburgh EH9 1LF, UK; 4 Usher Institute, Edinburgh Medical School, University of Edinburgh, Edinburgh EH16 4UX, UK; mils.lo@ed.ac.uk

**Keywords:** symbolic data analysis, nonlinear analysis, dispersion entropy, missing samples, outlier samples

## Abstract

Entropy quantification algorithms are becoming a prominent tool for the physiological monitoring of individuals through the effective measurement of irregularity in biological signals. However, to ensure their effective adaptation in monitoring applications, the performance of these algorithms needs to be robust when analysing time-series containing missing and outlier samples, which are common occurrence in physiological monitoring setups such as wearable devices and intensive care units. This paper focuses on augmenting Dispersion Entropy (DisEn) by introducing novel variations of the algorithm for improved performance in such applications. The original algorithm and its variations are tested under different experimental setups that are replicated across heart rate interval, electroencephalogram, and respiratory impedance time-series. Our results indicate that the algorithmic variations of DisEn achieve considerable improvements in performance while our analysis signifies that, in consensus with previous research, outlier samples can have a major impact in the performance of entropy quantification algorithms. Consequently, the presented variations can aid the implementation of DisEn to physiological monitoring applications through the mitigation of the disruptive effect of missing and outlier samples.

## 1. Introduction

With the advancement of physiological recording technology deployed across a broad spectrum of applications, from wearable devices to intensive care units, increased amounts of data are becoming available for analysis [1,2]. While derived information can aid medical decision making, leading to personalised and prompt treatments, the successful implementation of data analysis algorithms is limited by challenges arising from the quality of recorded data due to the increased amount of missing and outlier samples, which are common occurrences due to user movement, loose equipment attachment, and electromagnetic interference [3,4,5]. In the case of wearable devices, low data quality caused by missing and outlier samples can limit the prognostic effectiveness of the algorithms, while in the case of intensive care units it can be life threatening through the phenomenon of “alarm fatigue” [6,7]. That is, algorithms currently deployed in intensive care units display excessive amounts of false positive alarms, causing clinical staff to ignore alarms that are perceived as false even when they are accurate, due to “alarm fatigue,” thereby putting patients at risk [2,8].

Concurrently, entropy quantification algorithms have emerged as a prominent tool for the characterisation of the physiological state of individuals through the measurement of irregularities of physiological signal segments. Building upon the initial extension of entropy to information theory by Shannon [9], novel variations such as Approximate Entropy (ApEn) [10], Sample Entropy (SampEn) [11], Permutation Entropy (PEn) [12], Fuzzy Entropy (FuzzyEn) [13], and Dispersion Entropy (DisEn) [14] have been implemented as nonlinear indexes aiding disease diagnosis and prognosis. Examples of implementations include the use of ApEn for the investigation of abnormalities in respiratory function caused by panic disorders [15], the analysis of neonatal heart rate variability using SampEn for improved diagnosis of sepsis [16], the analysis of electroencephalogram (EEG) signals to track the state of consciousness of patients while under the effect of anaesthetic drugs using PEn [17], the application of FuzzyEn on surface electromyography (EMG) signals for the detection of motion [13], and the analysis of blood pressure signals to quantify the effect of aging in the reduction of the signal’s irregularity using DisEn [14]. However, while the performance of these algorithms is promising, it is important to ensure that they are robust to increased numbers of missing and outlier samples prior to their deployment.

The robustness of ApEn, SampEn, and FuzzyEn has been tested when analysing time-series containing missing samples, and the results indicate that, while the classification capacity of the algorithms can be preserved under certain conditions, the fluctuations of entropy values can be large, affecting the accuracy of the results extracted for each analysed signal segment [18]. Furthermore, recent research has provided new variations of SampEn leading to improved performance when analysing time-series with missing samples [19]. Concerning the effect of outliers, ApEn and SampEn have been tested, and the results indicate that outlier samples can disrupt the process of entropy quantification to a much greater extent than missing samples and should therefore be a key consideration when testing the robustness of respective algorithms [20,21].

This study aims to expand upon this research focusing on the DisEn algorithm due to its favorable performance characteristics such as increased discrimination capacity and low computation time [22,23]. The research presented in this manuscript focuses on:

- The quantification of the effect of missing and outlier samples on the performance of DisEn.
- The introduction of new variations of the DisEn algorithm to improve its performance when applied to time-series with missing and outlier samples.
- The assessment of the performance of the original algorithm and its variations, across different physiological datasets and under separate experimental setups defined by the percentage of missing or outlier samples and the degree to which these samples are grouped together or exist individually.

The article is structured in the following manner. The Methods section provides an overview of the DisEn algorithm and presents its algorithmic variations developed as part of this study. It continues by presenting the datasets used, the process for producing time-series containing missing and outlier samples, the metrics used for performance assessment, and the statistical analysis applied to the results of the designed experimental setups. The Results section provides a summary of the results of the implemented statistical tests, continues by presenting the performance of DisEn variations, for each physiological type separately, applied to time-series with missing samples, and closes with the respective performance for time-series with outlier samples. In the Discussion section, important insights from the study are reviewed, and performance patterns are examined and interpreted considering the interplay between normal samples and missing or outlier samples, the spectral characteristics of analysed time-series, and the operation of the respective DisEn variation. Finally, limitations of the current study are addressed, and opportunities for future work highlighted.

## 2. Methods

### 2.1. Dispersion Entropy (DisEn)

DisEn arises from Shannon entropy with the integration of symbolic dynamics for the development of an algorithm capable of quantifying the degree of irregularity of investigated signal segments with low computation time, while maintaining increased discrimination capacity [14]. The process followed by the algorithm for the analysis of a given univariate time-series $x_{j}\;\left(j=1,2,\ldots ,N\right)$ of length *N* is the following:

1. A first and optional step is the mapping of the time-series with a linear or non-linear mapping function. For the formulation of the majority of mapping functions, the mean and standard deviation of the time-series are computed and used.
2. A number of classes (*c*) is then mapped to the resulting signal by being distributed across its amplitude range. Each sample is allocated to the nearest respective class based on its amplitude. As a result, a classified signal $u_{j}\;\left(j=1,2,\cdots ,N\right)$ is retrieved.
3. With the classified signal defined, an embedding dimension (*m*) and a time delay (*d*) are set for the creation of multiple time-series, of length *m*, $u_{i}^{m,c}=\left\{u_{i}^{c},u_{i+d}^{c},\cdots ,u_{i+(m-1)d}^{c}\right\}$ for each i=1,2,⋯,N−(m−1)d. Each time-series $u_{i}^{m,c}$ is mapped to its respective dispersion pattern, with the number of potential dispersion patterns being $c^{m}$.
4. For each dispersion pattern, its relative frequency is obtained and used to calculate the DisEn value of the input time-series based on Shannon’s definition of entropy.

**Figure 1:**
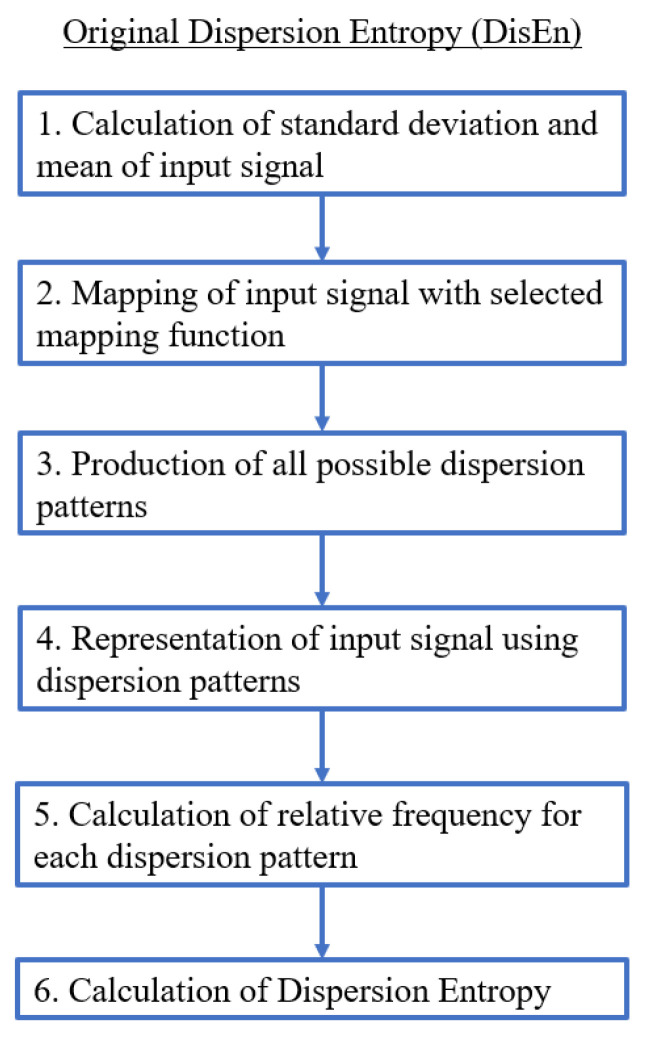
Algorithmic diagram of the original Dispersion Entropy (DisEn) algorithm.

Therefore, an input signal that could be described by a single dispersion pattern would have a minimum DisEn value as opposed to one requiring all possible patterns in equal probability, in which case it will have a maximum DisEn value. The minimum and maximum DisEn value range is defined by the parameter values chosen for the implementation of the algorithm. Figure 1 displays an algorithmic block diagram presenting the computational steps for the implementation of the original DisEn algorithm. Further details concerning the operation of the DisEn algorithm such as suggested mapping approaches, optimisation of parameter values, and performance evaluation are available in [22].

### 2.2. Dispersion Entropy Variations

The following variations of the algorithm are developed and tested for improved performance when used for the analysis of time-series that contain missing and outlier samples.

#### 2.2.1. Skip Sample Dispersion Entropy (SkipDisEn)

**Figure 2:**
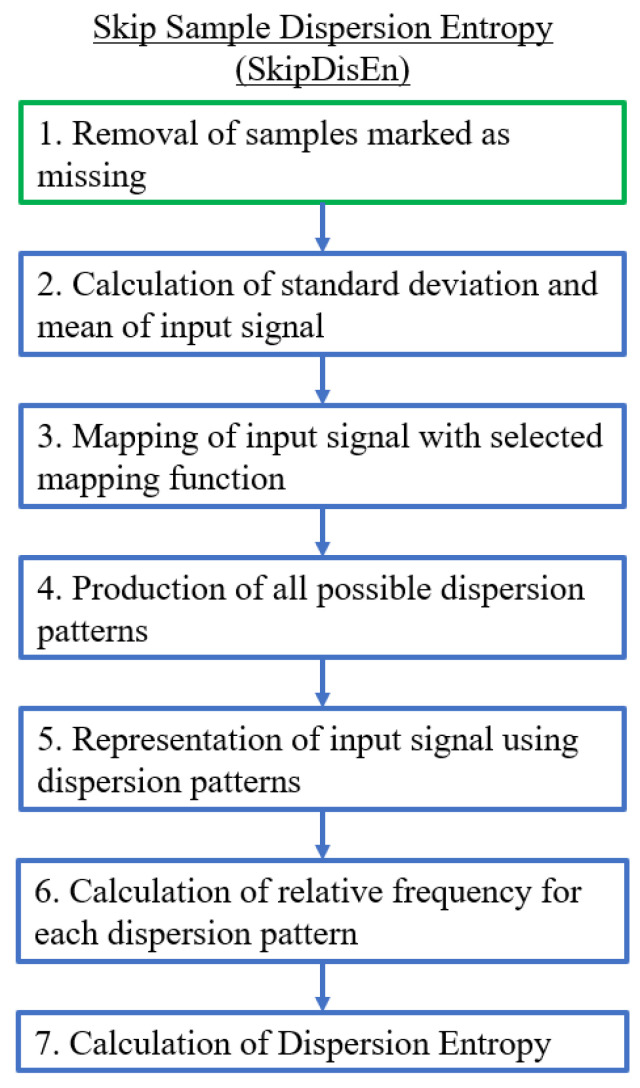
Algorithmic diagram of the Skip Sample Dispersion Entropy (SkipDisEn) variation. The added step is outlined in green.

The SkipDisEn variation removes samples marked as missing and connects the remaining samples in a continuous time-series based on the computational steps shown in Figure 2. This is the default approach followed in prior entropy quantification algorithms [16,18]; in this study, we are interested in assessing the effectiveness of the respective DisEn variation when applied on time-series with missing samples.

#### 2.2.2. Linearly Interpolated Dispersion Entropy (LinInterDisEn)

**Figure 3:**
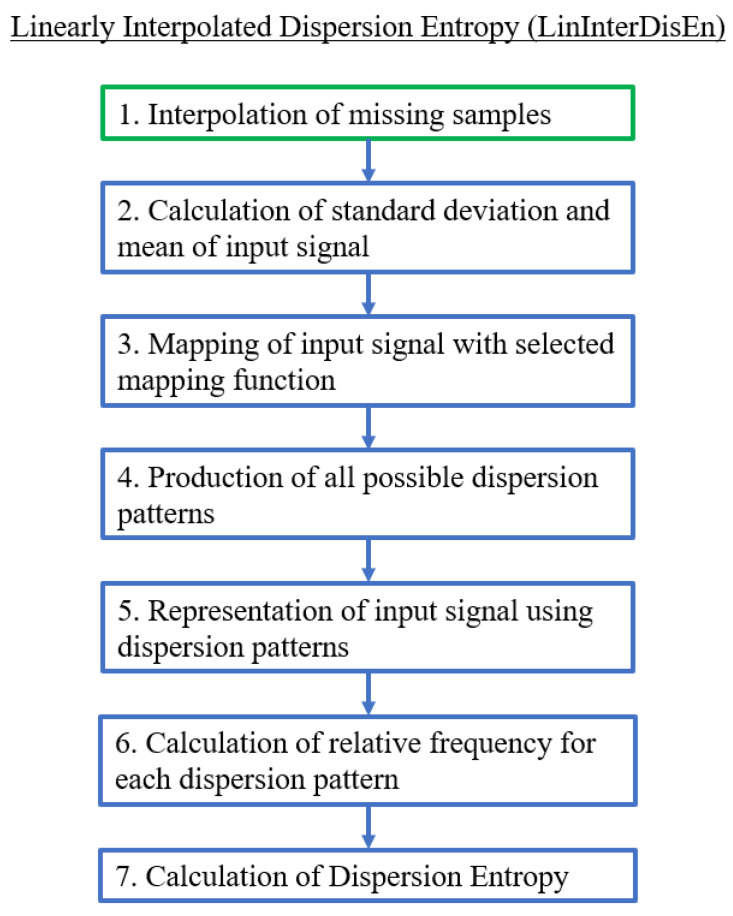
Algorithmic diagram of the Linearly Interpolated Dispersion Entropy (LinInterDisEn) variation. The added step is outlined in green.

The LinInterDisEn variation uses linear interpolation to replace samples tagged as missing based on the equation $y\left(x\right)=\frac{y_{0}\left(x_{1}-x\right)+y_{1}\left(x-x_{0}\right)}{x_{1}-x_{0}}$, where $y_{0}$, $y_{1}$ are the amplitudes, and $x_{0}$, $x_{1}$ are the locations of the nearest available samples. In this variation, linear interpolation is being implemented due to promising results of performance improvement for entropy quantification algorithms in previous research [19,24]. Similarly to SkipDisEn, this variation focuses on having improved performance when dealing with missing samples. Its computational steps are shown in Figure 3.

#### 2.2.3. Alternative Statistical Metrics Dispersion Entropy (AltMetDisEn)

**Figure 4:**
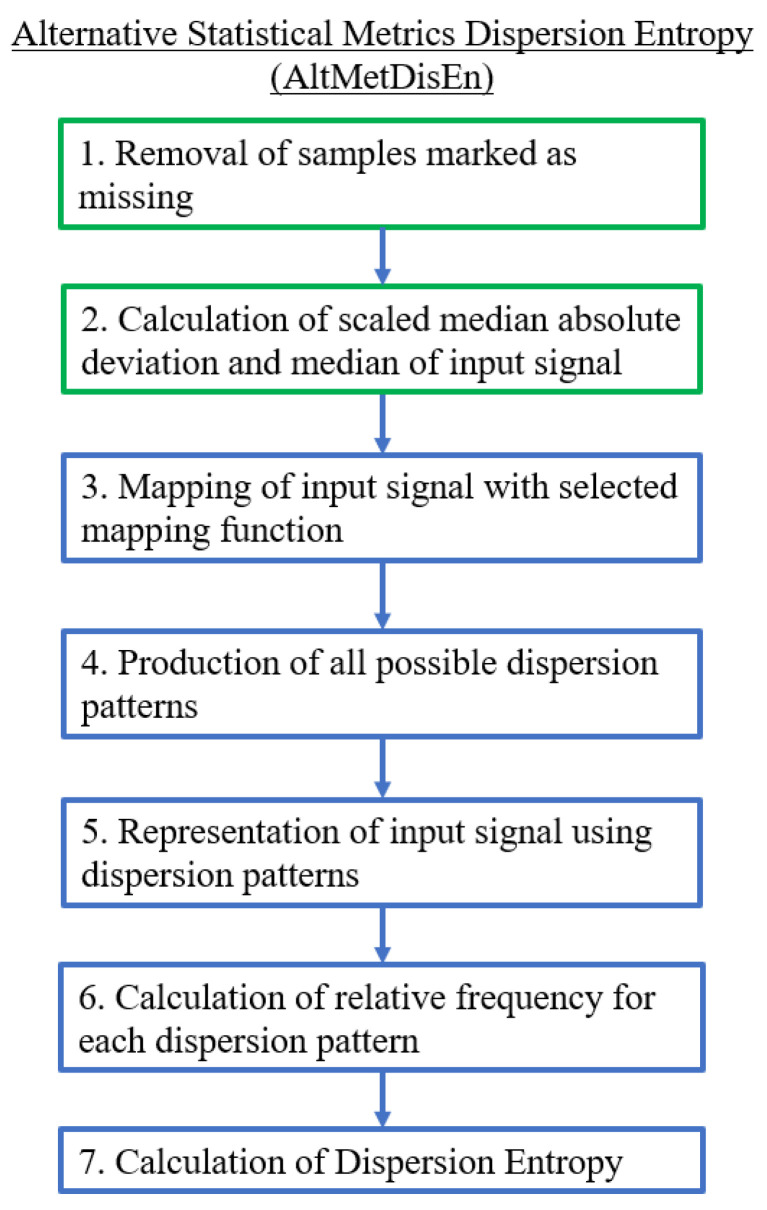
Algorithmic diagram of the Alternative Statistical Metrics Dispersion Entropy (AltMetDisEn) variation. The added and modified steps are outlined in green.

The AltMetDisEn variation uses alternative statistical metrics for the implementation of mapping functions. The originally used mean is replaced with a median and standard deviation is estimated using the median absolute deviation multiplied by the scaling factor of 1.4826 [25]. The new statistical metrics are chosen for their robustness to outliers in order to reduce the disruption of classes allocation due to the increases in the amplitude range of the input signals caused by outliers [26]. Furthermore, AltMetDisEn is modified in the same manner as SkipDisEn in order to skip any samples tagged as missing and is therefore expected to have an analogous performance on the analysis of time-series with missing samples. The algorithmic diagram of AltMetDisEn is shown in Figure 4.

#### 2.2.4. Dynamic Skip Sample Dispersion Entropy (DynSkipDisEn)

**Figure 5:**
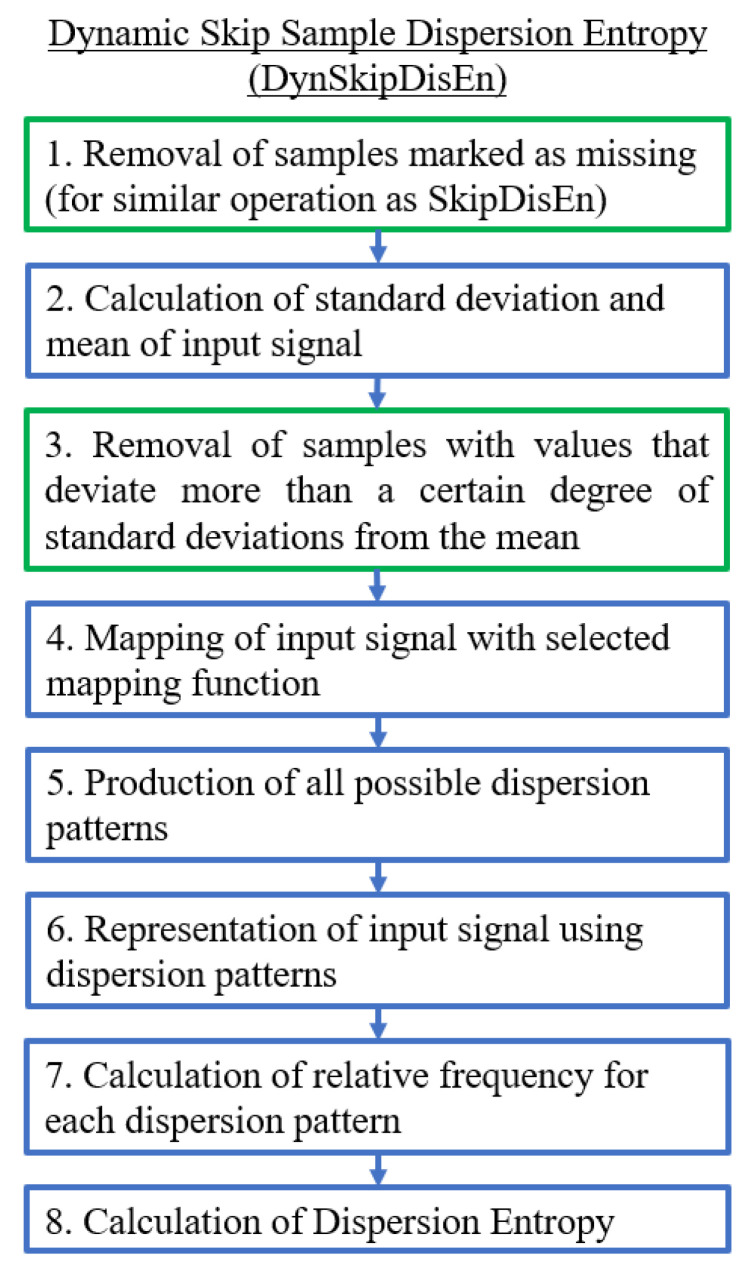
Algorithmic diagram of the Dynamic Skip Sample Dispersion Entropy (DynSkipDisEn) variation. The added and modified steps are outlined in green.

The DynSkipDisEn variation implements a dynamic skipping approach through the use of an additional parameter named cutoff. DynSkipDisEn aims at replicating the performance of SkipDisEn when applied to time-series with outlier samples by automatically discarding any samples with values that deviate more than a certain degree of standard deviations, defined by the cutoff parameter, from the mean of each analysed signal segment, as shown in Figure 5. Since the cutoff threshold of this algorithm is a scaled version of the standard deviation of its input signal segment, the effect of outlier samples in the calculated standard deviation should be taken into consideration when selecting the value of the cutoff parameter, as discussed in Section 4.5.

### 2.3. Experimental Datasets

Aiming to develop variations of the DisEn algorithm with robust performance across a spectrum of monitoring applications, the following physiological signals are chosen for the study.

Heart rate interval (RR) data are commonly monitored in a range of biometric applications from wearable devices to patient monitoring in intensive care units for monitoring the cardiovascular system of individuals [27,28]. The Fantasia Database [29] publicly available in Physionet [30] contains 40 electrocardiogram (ECG) recordings of healthy adults sampled at 250 Hz, while also providing the respective RR interval data, which are used for this study. In total, RR interval data of 20 young adults are chosen for analysis.

In addition, electroencephalograms (EEG) are chosen as a representative and commonly analysed signal for monitoring the nervous system of individuals. The EEG signals used for this study are the FP1-F7 channel recordings of differential signals sampled at 256 Hz of 13 selected individuals retrieved from the publicly available CHB-MIT Scalp EEG Database [30,31].

Finally, to measure the performance of the algorithm in monitoring the operation of the respiratory systems of individuals, the respiratory impedance (RI) signal is chosen. A total of 15 recordings are chosen by the publicly available BIDMC PPG and Respiration Dataset [30,32], each containing 8 minutes of impedance respiratory signal sampled at 125 Hz.

### 2.4. Generation of Disrupted Time-Series

To produce “disrupted” time-series, time-series containing missing (disruptedM) or outlier (disruptedO) samples, used for measuring the performance of different variations of the DisEn algorithm, the following processes are used. For the production of disruptedM time-series:

1. Extraction of ground truth DisEn values. Each original time series containing *N* points without missing samples is separated in non-overlapping windows of 360 samples each. The choice of window length is made with the aim to test algorithmic performance under the restriction of small sample lengths, which is considered one of the advantages of the original DisEn algorithm [22,33]. The original algorithm DisEn is used to calculate the ground truth DisEn value of each respective window.
2. Segmentation of time-series. Copies of the initial time-series are segmented in groups of 1–5 samples, as defined by the grouping factor *G*. The *G* factor values used are 1, 2, 3, 4, and 5 samples.
3. Marking of missing samples. Based on the percentage factor *P*, a percentage of segments are uniformly drawn from each time-series and their samples are marked as missing. The *P* factor values used in this study are 10%, 20%, 30%, 40%, and 50%.
4. Production of random variations. Finally, the above process is replicated 10 times for each combination of *P* and *G* values producing different random variations for each experimental setup.
5. Total number of disrupted time-series. As a result from each initial time series, 5×5×10=250 “disruptedM” versions are produced, and these are used to assess the performance of the DisEn algorithm variations.

The rationale of choice for the above setups of “disruption” is to acquire a clear perspective of how increases in the number of missing samples affect the performance of DisEn algorithms and whether that performance changes when these missing samples are distributed individually or clustered together in groups considering that both events are common in physiological monitoring applications.

For the production of “disruptedO” time-series, an almost identical process is used. However, in this case, the modified samples are not marked as missing; instead, their amplitude is replaced with a value outside the physiological range of the original signal. Similarly to previous experiments, testing the robustness of ApEn and SampEn to outliers [21], the amplitude of each outlier sample is obtained from a Gaussian distribution. We use a standard deviation of 0.5 and a mean defined separately for each physiological time-series based on the formula: outliermean=±4×max|amplitude|. Half of the modified samples are given a positive value, and half of them are given a negative value. For *G* factors higher than 1, all modified samples within a group share the same sign and value. The choice of setting the mean of the distribution to be the maximum absolute amplitude observed in the input time-series multiplied by a factor of 4 is made to ensure that outlier samples are outside the physiological range of the recorded signal, and at the same time simulate the limitation of the maximum amplitude of the recording equipment. A standard deviation of 0.5 is chosen to allow outlier values to vary, as it is expected, while at the same time not allow their range of values to spread within physiological range. Similarly to time-series with missing samples for each original time-series, 5×5×10=250 “disruptedO” versions are produced.

### 2.5. Performance Assessment

As mentioned in Section 2.4, the initial time series is separated in windows and the ground truth DisEn value, for each window, is computed and stored using the original DisEn algorithm. The same process takes place for each disrupted time-series, and the absolute percentage deviation is calculated using the ground truth DisEn value of a specific window versus the equivalent DisEn value calculated from the “disrupted” version of the same window. This assessment is applied to each physiological dataset separately. To summarise the performance of the selected algorithm for a setup of *P* and *G* values, a single mean absolute percentage deviation value is acquired and presented alongside its respective standard deviation. This is achieved by averaging across

1. the windows of each time-series,
2. the 10 different “disrupted” editions of each time series, and
3. the total number of time-series that have been chosen from the respective dataset records.

Furthermore, in the case of the AltMetDisEn variation, if the variation is applied to the original time-series, the calculated DisEn values will differ from those of the original DisEn algorithm. This occurs because the changes implemented in the AltMetDisEn variation occur prior to mapping the input signal with a selected mapping function and therefore have an effect even when no missing or outlier samples exist. To maintain consistency with the rest of the performance measurements, the values of the original DisEn algorithm are used as ground truth in the calculation of error percentages for the AltMetDisEn similarly to the rest of the variations. In order to measure the amount of error that occurs even when AltMetDisEn is applied to the original time-series due to the difference in DisEn values with the original DisEn as opposed to the error that occurs due to missing or outlier samples, the AltMetDisEn variation is also applied to each of the original time-series, and the mean absolute percentage deviation from the original DisEn values is calculated and reported in the respective parts of Section 3. Finally, for all variations of DisEn tested, including the original algorithm, the parameter values chosen are as follows:

- Embedding dimension: m=2 samples.
- Number of classes: c=6 classes.
- Mapping approach: logarithm sigmoid function.
- Time delay: 1 sample.
- Cutoff: 0.7 standard deviation (used only by DynSkipDisEn).

The parameter values are selected after consulting the respective literature [14,22] and considering that each input window used in our study has a length of 360 samples [33]. The MATLAB codes for the implementation of the algorithmic variations presented in this paper are publicly available at https://doi.org/10.5281/zenodo.3629475.

### 2.6. Statistical Testing

The following statistical analysis is applied for the error percentage distributions produced by DisEn variations during each experimental setup.

- Kolmogorov-Smirnov Test. Each separate distribution is standardised and compared to a standard normal distribution using a Kolmogorov-Smirnov test.
- Mann-Whitney *U* Test. Based on the results of the Kolmogorov-Smirnov test, a Mann-Whitney *U* test is chosen and applied to all distribution pairs produced within the same experimental setup to test whether the distribution of error percentages produced by one DisEn variation is significantly different from the distributions produced from the other variations tested under the same experimental setup.

## 3. Results

### 3.1. Summary of Statistical Testing Results

The results of the Kolomogorov-Smirnov test applied to all error percentage distributions after they have been standarised indicate that all distributions reject the null hypothesis at the 1% significance level. Therefore, their error percentages do not come from a Gaussian distribution.

The Mann-Whitney *U* test, which was chosen after taking into consideration the non-Gaussian nature of the distributions, indicates that out of the total 450 distribution pairs tested, 441 *U* tests reject the null hypothesis with a strict threshold of a *p*-value lower than $10^{-3}$. Actually, 97% of *p*-values are lower than $10^{-7}$. The nine error percentage distribution pairs that do not display statistically significant difference are signified in their respective sections that follow.

### 3.2. Experimental Setups for Time-Series with Missing Samples

The variations SkipDisEn, AltMetDisEn, and LinInterDisEn were tested on the three separate physiological datasets of RR, EEG, and RI time-series that have been modified to contain missing samples, as described in Section 2.4. Their performance was assessed under 25 different experimental configurations as defined by the percentage of missing samples, *P* factor, and the grouping of missing samples, *G* factor. The original version of the DisEn algorithm would return an invalid output if a single sample within the input time-series was marked as missing, resulting in very low performance when dealing with time-series containing missing samples. Therefore, in this part of our analysis, only the performances of new variations are presented and compared.

#### 3.2.1. Performance for RR Time-Series with Missing Samples

**Figure 6:**
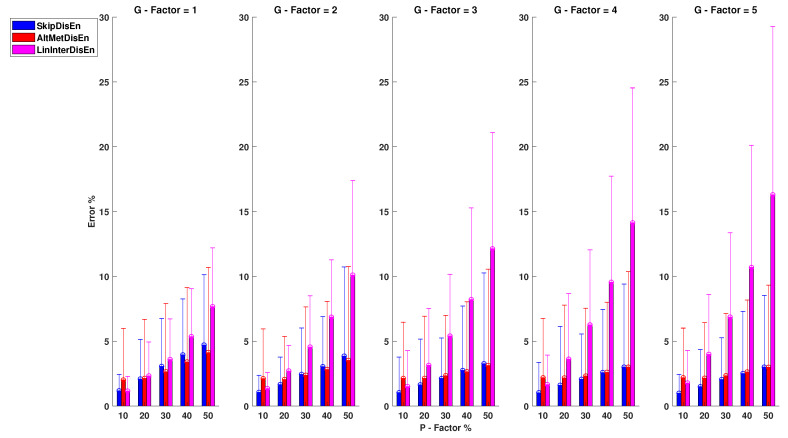
Performance of DisEn variations on heart rate interval (RR) time-series with missing samples. Mean and standard deviation of the percentage error are shown for each tested variation. The distribution pairs of SkipDisEn and AltMetDisEn for: P=40%, G=4; P=50%, G=4; P=40%, G=5; P=50%, G=5 do not display statistically significant difference based on the Mann-Whitney *U* test.

As shown in Figure 6, SkipDisEn and AltMetDisEn display similar performance when analysing RR time-series. The mean percentage error for SkipDisEn is within the range of 0.98 and 4.70%, with minimum values at P=10%, G=5 and maximum values at P=50%, G=1, respectively. The mean percentage error for AltMetDisEn is within the range of 2 and 4.12% observed at P=10%, G=1 and P=50%, G=1, respectively. Furthermore, the mean percentage deviation of the ground truth values for AltMetDisEn from the original ground truth values is calculated at 2.41% with a standard deviation of 1.23%.

LinInterDisEn displays a significantly higher average error rate within the range of 1.15 and 16.29% with minimum deviation at P=10%, G=1 increasing significantly especially in cases of “clustered” missing samples (higher *G*-Factor values) to reach a maximum average error rate of 16.29% observed at P=50%, G=5. The effect of “clustered” missing samples in the performance of LinInterDisEn is expected due to the reduced accuracy of synthetic samples produced using linear interpolation when a higher number of adjacent samples are missing.

Out of the 75 *U* test results retrieved in this group of experimental setups, 4 distribution pairs do not display statistically significant difference. These consist of the error percentage distributions acquired from the SkipDisEn and the AltMetDisEn variations for the experimental setups of:

- P=40%, G=4 with a *p*-value of 0.19.
- P=50%, G=4 with a *p*-value of 0.03.
- P=40%, G=5 with a *p*-value of 0.01.
- P=50%, G=5 with a *p*-value of 0.19.

#### 3.2.2. Performance for EEG Time-Series with Missing Samples

**Figure 7:**
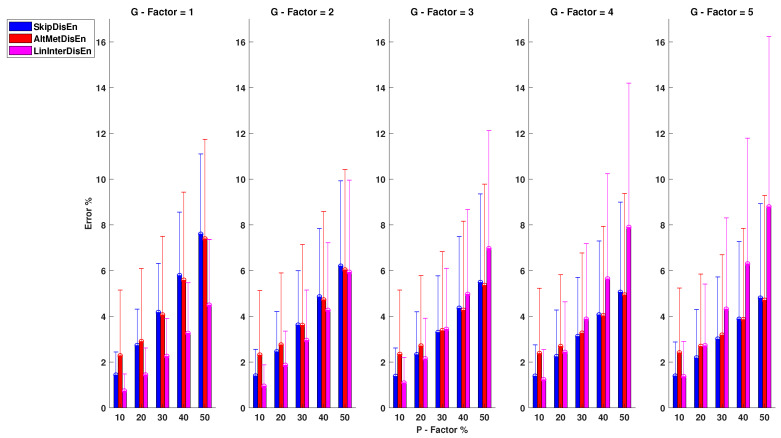
Performance of DisEn variations on EEG time-series with missing samples. Mean and standard deviation of the percentage error are shown for each tested variation. The distribution pairs of AltMetDisEn and LinInterDisEn for P=50%, G=2 and SkipDisEn and LintInterDisEn for P=10%, G=5 do not display statistically significant difference based on the Mann-Whitney *U* test.

As shown in Figure 7, SkipDisEn and AltMetDisEn maintain similar levels of performance. However, in this analysis, LinInterDisEn displays better performance for lower *P* and *G* factor values. SkipDisEn’s error is within the range of 1.38 and 7.59% observed at P=10%, G=3 and P=50%, G=1 and similarly AltMeDisEn’s error is within the range of 2.28 and 7.39% observed at P=10%, G=1 and P=50%, G=1, respectively. The mean absolute deviation of ground truth values of AltMetDisEn from the original ground truth values is calculated at 2.42% with a standard deviation of 0.60%.

LinInterDisEn achieves improved performance for lower values of *P*,*G* compared to SkipDisEn and AltMetDisEn. Its error is within the range of 0.74 and 8.79% observed at P=10%, G=1 and P=50%, G=5, respectively. With increases in the values of experimental factors, particularly that of *G*, its initially superior performance eventually drops to lower than that of the aforementioned variations in the analysis of EEG time-series.

From the 75 *U* tests retrieved from this experimental setup, only two do not display statistical significance: the *U* test between the error percentage distributions of AltMetDisEn and LinInterDisen for P=50%, G=2 with a *p*-value of 0.59 and the *U* test between SkipDisEn and LinInterDisEn distributions for P=10%, G=5 with a *p*-value of 0.01.

#### 3.2.3. Performance for RI Time-Series with Missing Samples

**Figure 8:**
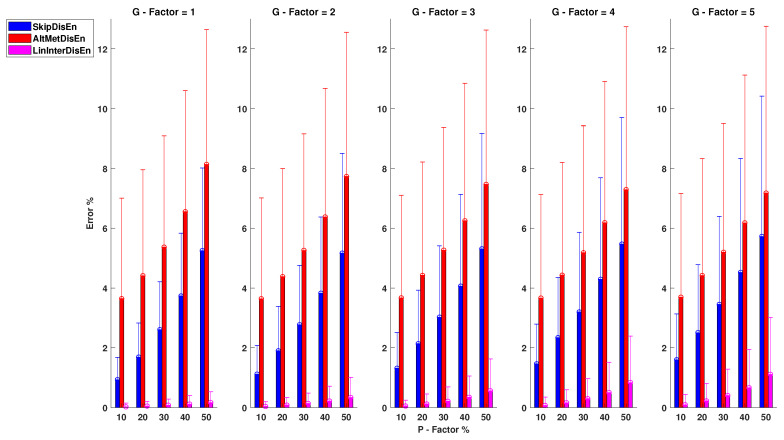
Performance of DisEn variations on RI time-series with missing samples. Mean and standard deviation of the percentage error are shown for each tested variation. All distribution pairs display a statistically significant difference based on the Mann-Whitney *U* test.

As shown in Figure 8, there is a significant performance difference between SkipDisEn and AltMetDisEn. SkipDisEn has a mean percentage error in the range of 0.93 and 5.72% for P=10%, G=1 and P=50%, G=5, respectively, while AltMetDisEn displays inferior performance with a mean percentage error in the range of 3.64 and 8.14% for P=10%, G=1 and P=50%, G=1. The mean absolute deviation between the ground truth values of AltMetDisEn and the original ones is calculated at 3.03% with a standard deviation of 1.47%. Both SkipDisEn and AltMetDisEn variations continue to follow a pattern of low error percentages that increase for higher *P*,*G* values across all tested physiological signals.

In the analysis of RI time-series, LinInterDisEn significantly outperforms SkipDisEn and AltMetDisEn, unlike in the case of RR and EEG time-series. Its mean percentage error is limited in the range of 0.03 and 1.11% with minimum and maximum values observed at P=10%, G=1 and P=50%, G=5, respectively. Unlike in the cases of SkipDisEn and AltMetDisEn, it can be seen that the performance of LinInterDisEn changes based on the physiological signals analysed. All *U* test results in this group of experimental setups indicate statistical significance between the distributions.

### 3.3. Experimental Setups for Time-Series with Outlier Samples

In contrast with the case of missing samples, the original version of the DisEn algorithm returns a valid DisEn value when applied to a window containing multiple outlier samples. Therefore, in this part of the study, the original version of the DisEn algorithm is used and its performance results are reported providing a starting point for measuring the effects of outliers on the calculation of DisEn values. The original DisEn algorithm and its AltMetDisEn and DynSkipDisEn variations were tested on the RR, EEG, and RI datasets under the same experimental configurations for factors *P* and *G* described in Section 3.2 and using the same values for the DisEn parameters as defined in Section 2.5. In this experimental setup, the analysed time-series have been modified to contain outlier samples outside the physiological range of each signal as described in Section 2.4.

#### 3.3.1. Performance on RR Time-Series with Outlier Samples

**Figure 9:**
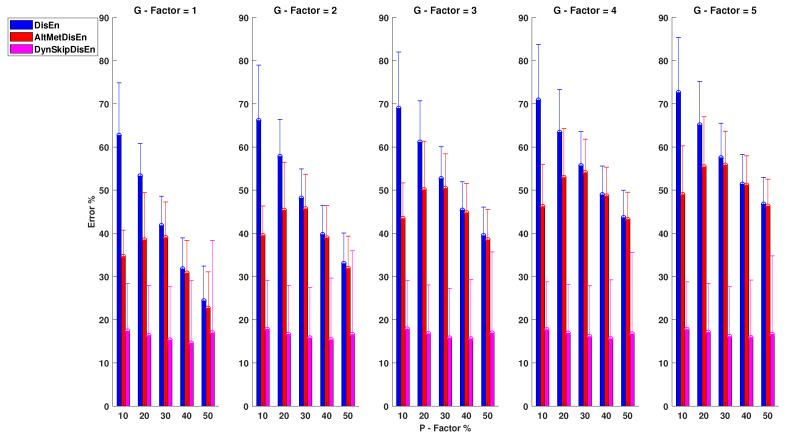
Performance of DisEn variations on RR time-series with outlier samples. Mean and standard deviation of the percentage error are shown for each tested variation. The distribution pair of the original DisEn and AltMetDisEn distributions for P=40%, G=5 does not display a statistically significant difference based on the Mann-Whitney *U* test.

Figure 9 shows that the original DisEn displays poor performance on the analysis of RR time-series, especially for lower values of the *P* factor. Its mean absolute error is in the range of 24.35 and 72.58% for the configurations P=50%, G=1 and P=10%, G=5, respectively. AltMetDisEn displays improved performance in the cases of low *P* values; however, for higher *P* values, its performance is similar to that of the original DisEn. Its percentage error is in the range of 22.64 and 55.78% observed at P=50%, G=1 and P=30%, G=5, respectively. DynSkipDisEn achieves the best performance with an error percentage in the range of 14.58 and 17.84% for P=40%, G=1 and P=10%, G=3. For the original DisEn and the AltMetDisEn variation, a certain amount of performance improvement is noticed as the percentage of outlier samples increases, which indicates that a deeper analysis on the effect of outlier values on the performance of DisEn is required. This is discussed in Section 4.4 of the study.

The only distribution pair that does not display a statistically significant difference for this group of experimental setups consists of the original DisEn and AltMetDisEn distributions for P=40% and G=5 with a *p*-value of 0.20.

#### 3.3.2. Performance for EEG Time-Series with Outlier Samples

**Figure 10:**
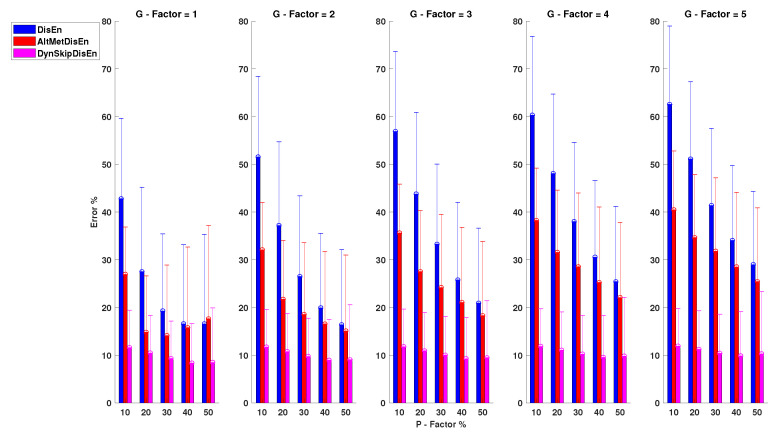
Performance of DisEn variations on EEG time-series with outlier samples. Mean and standard deviation of the percentage error are shown for each tested variation. All distribution pairs display statistically significant difference based on the Mann-Whitney *U* test.

In the case of EEG time-series, the performance of all variations seems to improve compared to RR time-series. As shown in Figure 10, the original DisEn displays an error rate in the range of 16.37 and 62.55% for P=50%, G=2 and P=1, G=5. AltMetDisEn achieves an improved performance for lower P and G values with a percentage error rate in the range of 14.11 and 40.47% for P=30%, G=1 and P=10%, G=5. Once more, the best performance is achieved by DynSkipDisEn with a percentage error limited in the range of 8.34 and 11.89% for P=40%, G=1 and P=10%, G=5. All distribution pairs for this group of experimental setups display a statistically significant difference.

#### 3.3.3. Performance RI Time-Series with Outlier Samples

**Figure 11:**
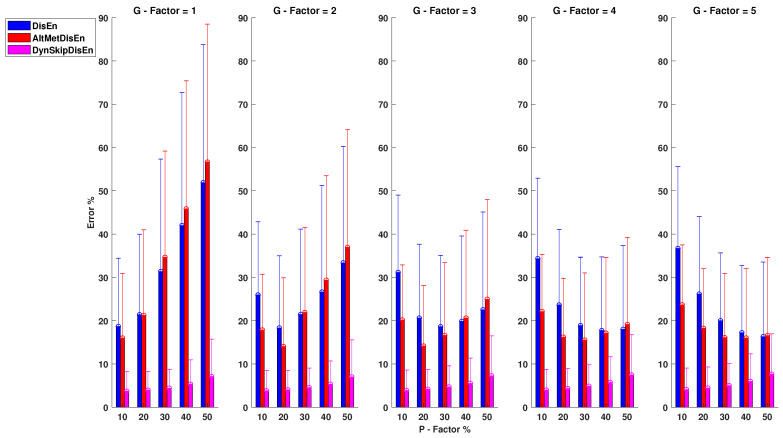
Performance of DisEn variations on RI time-series with outlier samples. Mean and standard deviation of the percentage error are shown for each tested variation. The distribution pairs of the original DisEn and AtlMetDisEn for P=50%, G=5 and AltMetDisEn and DynSkipDisEn for P=50%, G=5 do not display statistically significant differences based on the Mann-Whitney *U* test.

As shown in Figure 11, when applied to RI time-series, the original DisEn algorithm percentage error is in the range of 16.31 and 51.89% for P=50%, G=5 and P=50%, G=1. The AltMetDisEn performance shows a percentage error range of 14.02–56.65% for P=20%, G=2 and P=50%, G=1, respectively. DynSkipDisEn achieves a significantly improved performance with a percentage error limited in the range of 3.70–7.65% for P=10%, G=1 and P=50, G=5. For this group of experimental setups, two distribution pairs do not display any statistically significant difference. These consist of the original DisEn and the AltMetDisEn distributions for P=50, G=5 with a *p*-value of 0.01 and the AltMetDisEn and DynSkipDisEn distributions for P=50, G=5 with a *p*-value of 0.01.

### 3.4. Computation Time

To ensure that the variations presented and tested in this study preserve the low computation time of the original DisEn algorithm [22], we measured their computation time for the analysis of time-series with a length of 360 and with 9000 samples on randomly selected signal segments from all three physiological datasets, and the results are presented in Table 1 and Table 2, respectively. The computations are carried out using a PC with Intel(R) Core(TM) i7-8750H CPU @ 2.2 GHZ, 16 GB RAM running MATLAB R2018b. The computation time of the original DisEn algorithm is measured when applied to randomly selected segments of the original time-series, SkipDisEN and LinInterDisEn are applied to disruptedM time-series, while the AltMetDisEn and DynSkipDisEn are applied to disruptedO time-series. As the results indicate, no significant difference in the computation time is noted across the algorithmic variations apart from a small expected increase in the case of the LinInterDisEn variation observed at the signal segments with a length of 9000 samples due to the additional linear interpolation mechanism introduced.

## 4. Discussion

As part of this study, novel variations of the DisEn algorithm are introduced to improve its performance when applied to time-series with missing and outlier samples. Time-series from three different physiological signals— RR, EEG, and RI —are modified to produce multiple variations of time-series containing missing samples (disruptedM) and time-series containing outlier samples (disruptedO). Each produced variation of disruptedM and disruptedO time-series corresponds to a different experimental setup in order to assess the performance of algorithmic variations under different percentages of missing or outlier samples and under different degrees of grouping of these samples. The results of our analysis indicate that, while low-data quality, especially when it arises from artifactual outlier samples, can cause disruption in the entropy quantification mechanisms of the DisEn algorithm, significant improvements in its performance can be achieved with corresponding modifications.

### 4.1. Differences in the Effect of Missing versus Outlier Samples

An initial finding of the study concerns the effectiveness of DisEn when applied to disruptedM time-series versus its limited performance during the analysis of disruptedO time-series. In the case of disruptedM time-series, the SkipDisEn variation, which requires minimal modification of the initial DisEn algorithm, is capable of achieving a mean percentage error that remains lower than 7.6% across all examined physiological signals, even when up to 50% of the original samples are missing. Furthermore, the LinInterDisEn variation’s performance can surpass that of SkipDisEn, as shown in the analysis of disruptedM RI time-series. However, it is important to consider that LinInterDisEn’s performance is significantly affected by the spectral characteristics of the investigated signal and should therefore only be used when the respective information is available.

On the other hand, from the disruptedO time-series results, we can verify that, in the case of DisEn, similarly to ApEn and SampEn [20,21], outlier samples have a much more disruptive effect than missing samples. Taking into consideration the effectiveness of SkipDisEn in acquiring viable DisEn values, we recommend when possible to label outlier samples as missing in order to achieve a performance close to that observed in the analysis of disruptedM time-series. However, when the removal of all outlier samples is not guaranteed, the DynSkipDisEn variation is recommended. DynSkipDisEn is designed to tackle the disruption of the class allocation process, through the removal of samples that deviate from the mean more than a certain degree of standard deviations, as defined by the additional cutoff parameter.

### 4.2. Effect of Signal’s Spectral Characteristics on the Performance of LinInterDisEn

As shown in Section 3.2, the performance of LinInterDisEn is primarily affected by two factors: the clustering of missing samples on the analysed time-series, controlled by the *G* factor of the defined experimental setups, and the spectral characteristics of the physiological signal analysed. The clustering of missing samples has an expected negative effect on the performance of LinInterDisEn due to the reduced quality of synthetic samples produced using linear interpolation when a larger amount of adjacent samples are missing.

Furthermore, the spectral characteristics of the physiological signal analysed are expected to affect the performance of the linear interpolation mechanism when considering that RR time-series contain more high-frequency components [34], leading to rapid fluctuations in the amplitude of the signal that are harder to estimate using linear interpolation. RI time-series are dominated primarily by low-frequency components [35,36], leading to a larger number of linear signal segments that can be more accurately estimated. Finally, EEG time-series fall in between with a significant amount of high-frequency components [37], leading to amplitude fluctuations that can challenge the LinInterDispEn algorithm, especially in experimental setups with high *P* and *G* factor values.

### 4.3. Standard Deviations of Performance Measurements

The standard deviation values recorded throughout the presented experimental setups signify fluctuations in the performance of each tested variation on a window-by-window basis. This deviation occurs primarily due to two factors, the first one being that the disrupted time-series were formulated by introducing missing and outlier samples on the original time-series randomly, at the entire length of the time-series in order to more realistically simulate the phenomenon instead of equally distributing them across each window. Therefore, some windows would have more missing or outlier samples than others, leading to inevitable fluctuations in the tested performance.

However, the second factor that leads to increased standard deviation values of the mean performance error is the small sample length of the analysed windows. An important advantage of the original DisEn algorithm is its capacity to acquire valuable insights, even when applied to time-series windows with small sample lengths [22,33]; for that reason, we chose to test the performance of the original DisEn algorithm and its variations using 360 samples per window, which is a considerably smaller sample length than what was commonly used in similar studies concerning the performance of ApEn and SampEn when applied to time-series containing missing and outlier samples [18,19,21]. Considering the observed fluctuations in the algorithmic performance recorded in our study, we recommend that, for field applications where the DisEn value of each window is considered individually, a larger sample length is used when the analysed time-series are expected to contain missing and outlier samples.

### 4.4. Effect of Outlier Sample Percentage across Physiological Signals

**Figure A1:**
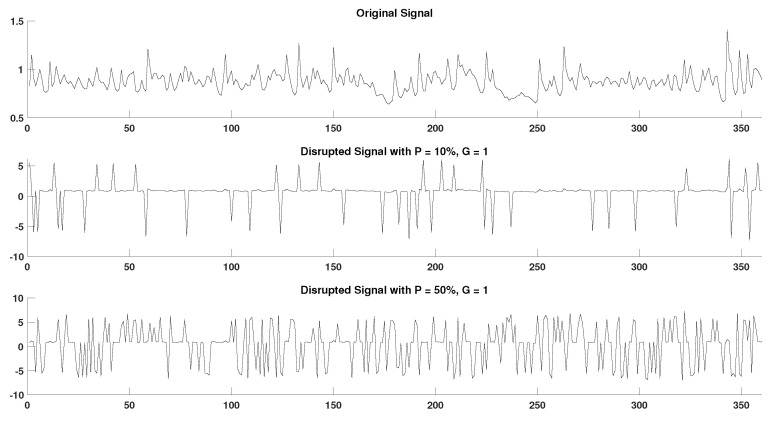
Original and disrupted signal segments of RR in support of Section 4.4.

**Figure A2:**
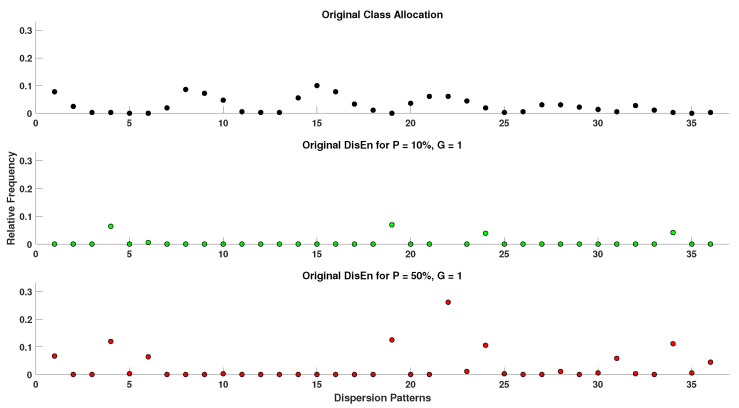
Original versus disrupted dispersion patterns of RR using the original DisEn algorithm in support of Section 4.4.

**Figure A3:**
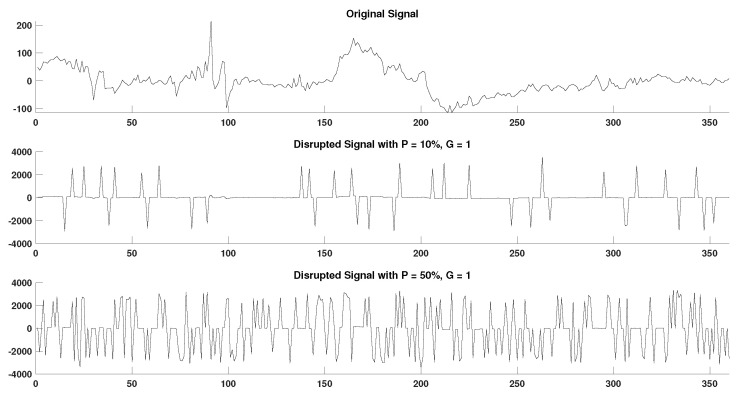
Original and disrupted signal segments of EEG in support of Section 4.4.

**Figure A4:**
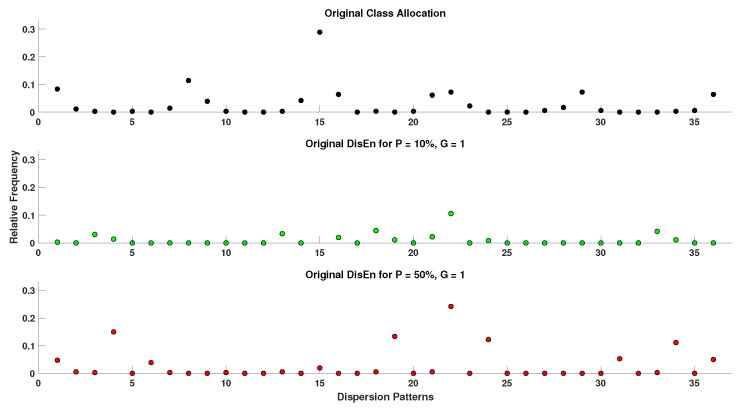
Original versus disrupted dispersion patterns of EEG using the original DisEn algorithm in support of Section 4.4.

**Figure A5:**
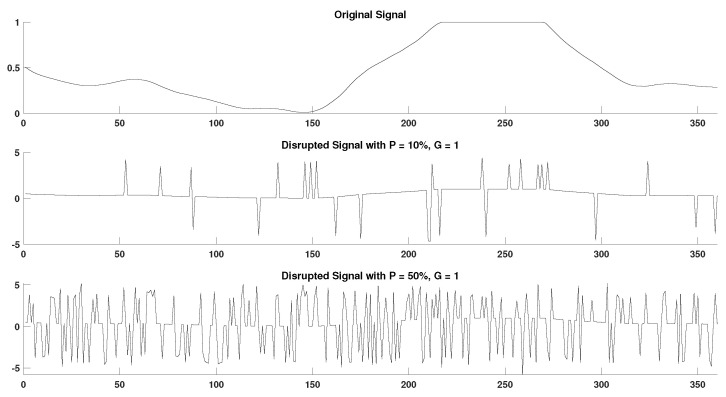
Original and disrupted signal segments of RI in support of Section 4.4.

**Figure A6:**
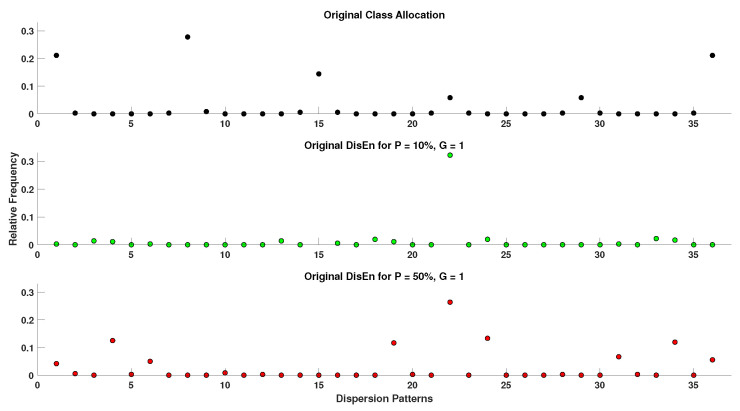
Original versus disrupted dispersion patterns of RI using the original DisEn algorithm in support of Section 4.4.

In order to acquire a better perspective on the effect of outlier samples in the performance of DisEn variations across different physiological signals, it is important to consider the mechanism through which outlier samples disrupt the DisEn calculation process. As mentioned in Section 2.1, during the second operational step of DisEn, a number of classes (c=6 in our experiments) are allocated across the amplitude range of the mapped input signal. With the introduction of outlier samples, this range expands significantly, resulting in fewer classes being allocated within the range of the original signal. Instead, the majority of classes are allocated in the extended amplitude range. As a result, amplitude dynamics existing in the original signal that would previously be represented using multiple dispersion patterns are now classified under a single dispersion pattern category, leading to a much lower output DisEn value. This phenomenon is shown in Figure A1, Figure A2, Figure A3, Figure A4, Figure A5 and Figure A6 (Appendix A), where examples of disrupted dispersion patterns for P=10%, G=1 are shown in green, and those for P=50%, G=1 are shown in red.

For high percentages of outlier samples, new dispersion patterns arise, and these do not represent physiological dynamics that occur within the original samples of the time-series but instead represent the amplitude dynamics that occur between original and outlier samples. This can lead to an increase in the irregularity of the input signal and therefore to an increase in the calculated DisEn value for disruptedO time-series with high *P* factor values. This is an important phenomenon to consider during the analysis of the performance of DisEn variations when tested on disruptedO time-series.

It is observed that, in the case of RR and EEG disruptedO time-series, with the effect being more prevalent in the case of RR, the performance of the original DisEn and the AltMetDisEn is actually increasing as the percentage of outlier samples increases, which at first can seem counterintuitive. However, in the case of RI time-series, the performance of DisEn variations does not follow a clear pattern. Taking into consideration the existence of rapid amplitude fluctuations in RR and EEG time-series, as opposed to the RI time-series, which contain primarily gradual changes in amplitude, as discussed in Section 4.2, the number of unique dispersion patterns used to describe each window of the original time-series is expected to be higher for RR followed by EEG and then by RI time-series; therefore, their respective DisEn values are expected to follow a similar pattern.

As mentioned previously for small values of the *P* factor, the mean DisEn value drops significantly in all three cases of physiological signals due to the time-series apparently becoming more regular as shown in green in Figure A1, Figure A2, Figure A3, Figure A4, Figure A5 and Figure A6. As the outlier percentage increases, more dispersion patterns are introduced, as shown in red in Figure A1, Figure A2, Figure A3, Figure A4, Figure A5 and Figure A6, in order to describe the now multiple amplitude fluctuations that occur between normal and outlier samples. Consequently, the DisEn values of all three physiological signals increase for higher *P* factor values. In the cases of RR and EEG time-series, the increase in DisEn values that occurs brings them closer to their respective ground truth values, resulting in the performance “increase” observed in Section 3.3.1 and Section 3.3.2. Therefore, this increase in performance is not achieved due to an internal mechanism of the algorithm but rather from the acquisition of DisEn values closer to the ground truth arising from amplitude fluctuations occurring between outlier and normal samples.

In the case of the original RI time-series, their corresponding DisEn are lower values compared to those for RR and EEG time-series. Therefore, increases in DisEn values for disruptedO time-series occurring from the aforementioned phenomenon do not necessarily bring the calculated DisEn values closer to the ground truth; therefore, algorithmic performance does not follow a pattern similar to that of RR and EEG disruptedO time-series.

Finally, in the case of DynSkipDisEn, the calculation of DisEn is not affected significantly by dispersion patterns arising from the interaction between original and outlier samples due to the significant amount of outlier samples that are removed.

### 4.5. Setting the Cutoff Parameter of DynSkipDisEn

When setting the value of the cutoff parameter for the DynSkipDisEn variation, it is important to balance two opposing sources of error. A high cutoff value, e.g., close to 2 standard deviations from the mean, can allow an extensive amount of outliers within the range of samples analysed by the algorithm, leading to a significant reduction in its performance. On the other hand, a strict low cutoff value can lead to the false positive removal of valid samples. Considering the more disruptive nature of outliers as opposed to that of missing valid samples, a more conservative approach towards choosing lower values for the cutoff parameter is recommended when considering multiple options. Therefore, when setting the value of the cutoff, the quality of the data to be analysed should be taken into consideration when corresponding information is available.

**Figure A7:**
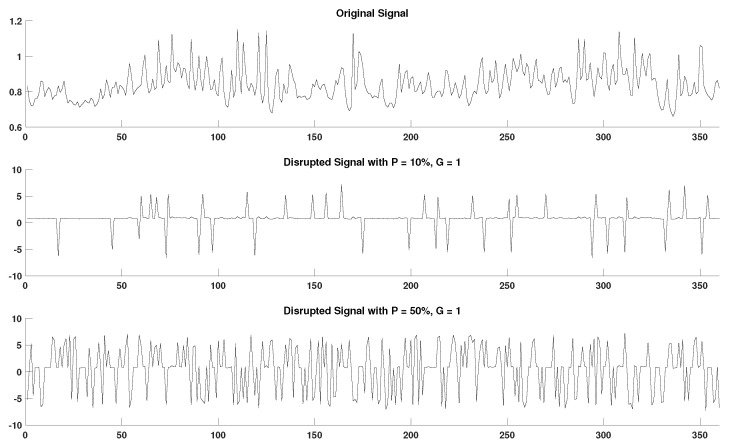
Original and disrupted signal segments of RR in support of Section 4.5.

**Figure A8:**
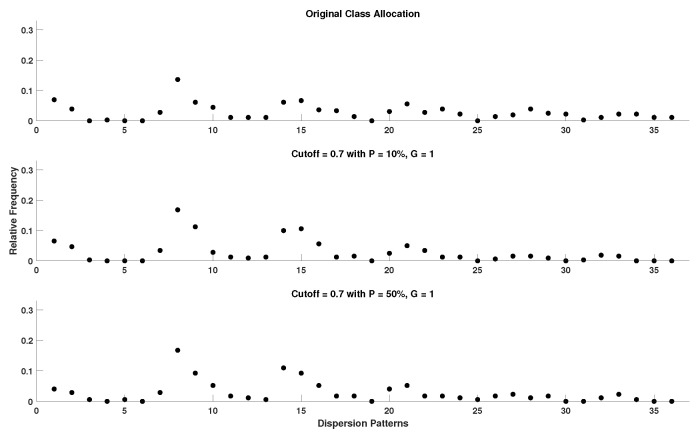
Original versus disrupted dispersion patterns of RR using DynSkipDisEn with a cutoff = 0.7 in support of Section 4.5.

**Figure A9:**
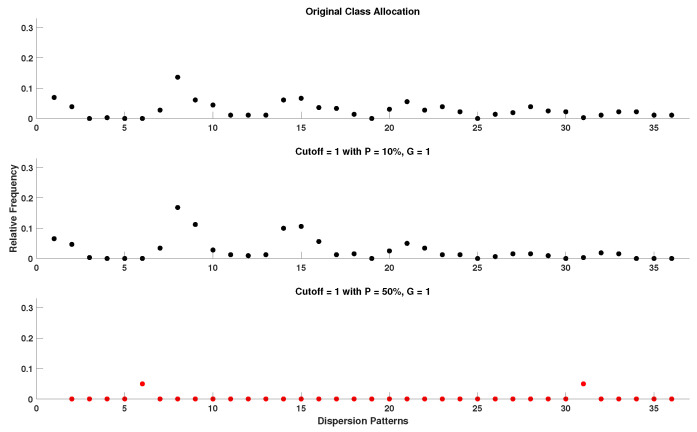
Original versus disrupted dispersion patterns of RR using DynSkipDisEn with a cutoff = 1 in support of Section 4.5.

**Figure A10:**
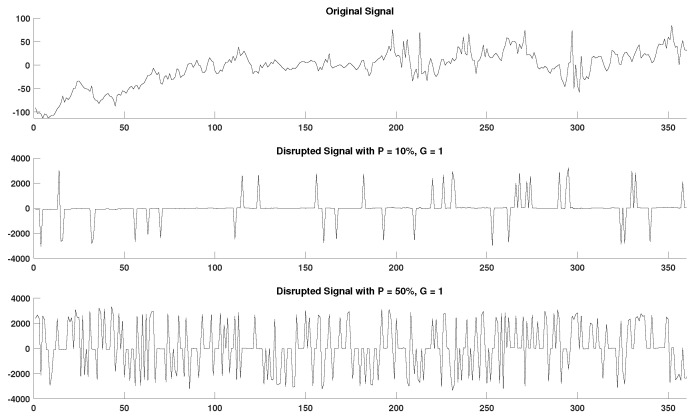
Original and disrupted signal segments of EEG.

**Figure A11:**
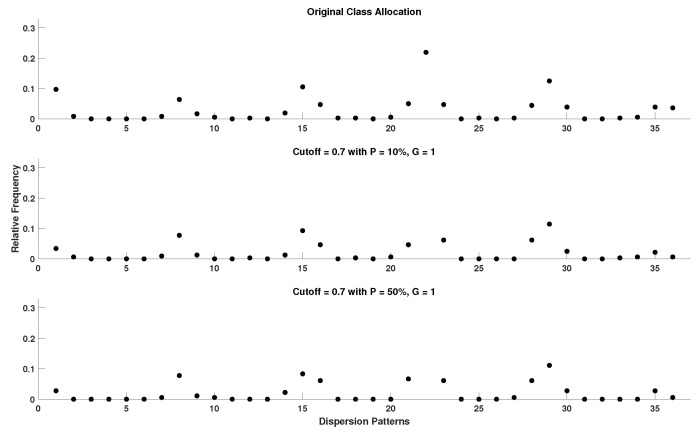
Original versus disrupted dispersion patterns of EEG using DynSkipDisEn with a cutoff = 0.7 in support of Section 4.5.

**Figure A12:**
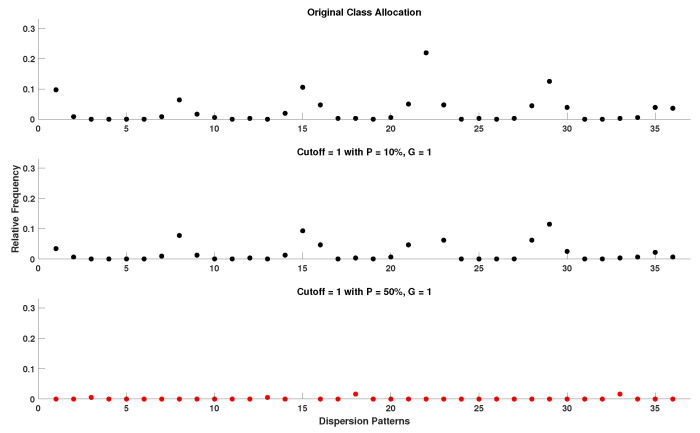
Original versus disrupted dispersion patterns of EEG using DynSkipDisEn with a cutoff = 1 in support of Section 4.5.

**Figure A13:**
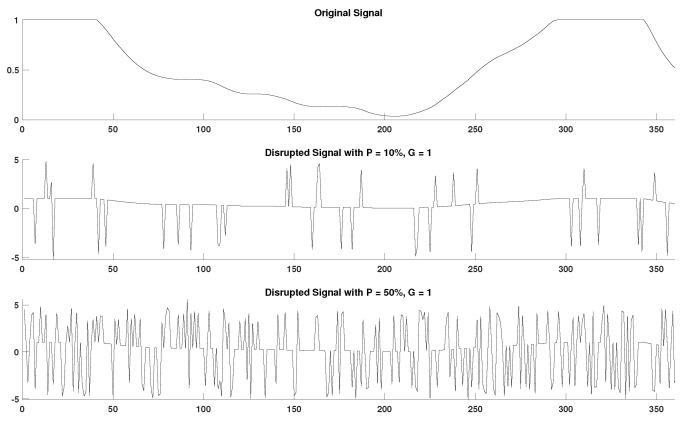
Original and disrupted signal segments of RI in support of Section 4.5.

**Figure A14:**
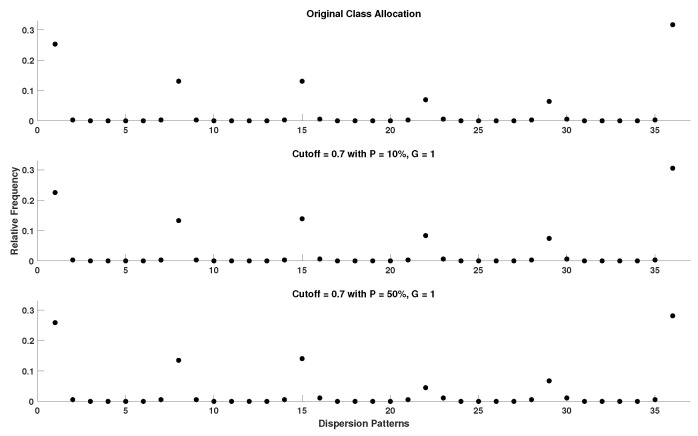
Original versus disrupted dispersion patterns of RI using DynSkipDisEn with a cutoff = 0.7 in support of Section 4.5.

**Figure A15:**
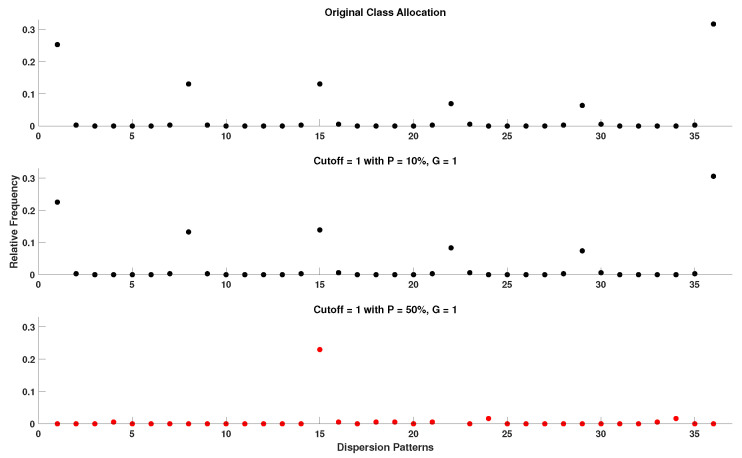
Original versus disrupted dispersion patterns of RI using DynSkipDisEn with a cutoff = 1 in support of Section 4.5.

Within the scope of this study, the cutoff parameter is set to the strict value of 0.7 standard deviations from the mean due to the high percentage of outlier samples introduced in the majority of our experimental setups. Furthermore, due to the range of values that outliers can cover, those with values further from the physiological range increase the calculated standard deviation of the input window, while those with values closer to physiological range have a higher probability of passing through the cutoff threshold, making a strict cutoff value a necessity. As shown in Figure A7, Figure A8, Figure A9, Figure A10, Figure A11, Figure A12, Figure A13, Figure A14 and Figure A15 (Appendix A), when comparing the capacity of DynSkipDisEn with a cutoff of 0.7 versus a cutoff of 1 to reconstruct the class allocation pattern of the original signal from its disruptedO versions, having a strict cutoff value of 0.7 leads to significantly improved performance, especially in the cases of higher outlier percentages where a cutoff of 1 leads to highly disrupted class allocation patterns. However, for applications where DynSkipDisEn is combined with preprocessing for the removal of outlier samples, we recommend a higher cutoff between 1 and 2, since in that case the percentage of remaining outliers in the time-series should be significantly lower, and a higher cutoff value would therefore provide improved performance.

### 4.6. Limitations of Current Study and Future Work

As indicated by the results of our study, the spectral characteristics of the investigated physiological signal have a direct effect on the performance of DisEn and its variations. Therefore, while the physiological signals used in our study—RR, EEG and RI—are commonly used for health monitoring applications, the study can be expanded to verify our observations in additional physiological signals such as EMG, blood pressure, and potentially intracranial pressure signals. Therefore, we suggest that similar experimental setups are used to assess the performance of DisEn variations prior to their deployment in respective applications.

Furthermore, the DisEn algorithm can be implemented using a wide variety of mapping functions. Within the scope of this study, the logarithm sigmoid function is used due to its successful implementation in previous studies of physiological signal analysis [22,33]. However, as mentioned in Section 4.4, outlier samples tend to disrupt the process of class allocation, which follows the mapping of the original time-series with the chosen mapping function. It would therefore be valuable to expand the study on measuring the robustness of different mapping functions to outliers such as the normal cumulative distribution function. Consequently, when optimising a DisEn variation for a specified implementation, the mapping function should be chosen by considering both the spectral characteristics of the input signal and the mapping function’s robustness to outlier samples.

Furthermore, while DynSkipDisEn is a promising variation trying to automatically remove outlier samples, there are two points that should be taken into consideration. The first one is that, even if the samples of the original time-series follow a Gaussian distribution, the existence of outliers will change the distribution in a non-Gaussian form, this should be taken into consideration, since it will affect the calculated mean and standard deviation based on which the DynSkipDisEn filters the samples of the input window. Finally, as suggested in Section 4.5, the correct choice of value for the cutoff parameter should consider the amount of outliers located in the analysed time-series. This information might not be available in certain applications. When this is the case, we recommend the choice of a relatively low cutoff value, considering the more disruptive nature of outliers when compared to missing samples.

## 5. Conclusions

This study investigates the effect of missing and outlier samples in the operation of DisEn and presents algorithmic variations to minimise their effect and improve its performance. The results indicate that the effect of missing samples can be effectively reduced with the addition of a skipping step in the operations of DisEn, while linear interpolation can further improve its performance when operating on time-series containing primarily low-frequency components. Outlier samples affect to a larger extent the performance of DisEn by disrupting the amplitude range during the class allocation step of the algorithm. A significant mitigation of the disruptive effect of outliers is achieved with the introduction of a cutoff parameter in the DynSkipDisEn variation. The presented DisEn variations operate using information only from within the signal segment that is used as input at a time to allow for a real-time entropy quantification process. However, upon availability, information concerning a time-series’ dominant frequency components and estimations of missing and outlier samples’ percentages can aid in the selection of the appropriate DisEn variation and the optimisation of its parameter values. We hope that the insights and algorithmic variations presented in this study will aid the implementation of DisEn in physiological monitoring applications.

## Figures and Tables

**Table 1:** Computation time in milliseconds for the signal segments of 360 samples.

	RR	EEG	RI
DisEn	1.6	1.5	1.9
SkipDisEn	1.5	1.4	1.9
AltMetDisEn	1.7	1.8	1.9
LinInterDisEn	1.6	1.7	2
DynSkipDisEn	1.5	1.4	1.5

**Table 2:** Computation time in seconds for the signal segments of 9000 samples.

	RR	EEG	RI
DisEn	2.1	2.3	2.3
SkipDisEn	2.4	2.5	2.2
AltMetDisEn	2.6	2.8	2.7
LinInterDisEn	3.1	3.3	3.2
DynSkipDisEn	2.5	2.5	2.6

## Abbreviations

The following abbreviations are used in this manuscript:

ApEn	Approximate Entropy
SampEn	Sample Entropy
PEn	Permutation Entropy
FuzzyEn	Fuzzy Entropy
EMG	Electromyography
DisEn	Dispersion Entropy
DisEn	Skip Sample Dispersion Entropy
LinInterDisEn	Linearly Interpolated
AltMetDisEn	Alternative Statistical Metrics Dispersion Entropy
DynSkipDisEn	Dynamic Skip Sample Dispersion Entropy
RR	Heart rate interval
ECG	Electrocardiogram
EEG	Electroencephalogram
RI	Respiratory Impedance

## References

[1] Witt D.R., Kellogg R.A., Snyder M.P., Dunn J. (2019). Windows into human health through wearables data analytics. Curr. Opin. Biomed. Eng..

[2] Paine C.W., Goel V.V., Ely E., Stave C.D., Stemler S., Zander M., Bonafide C.P. (2016). Systematic Review of Physiologic Monitor Alarm Characteristics and Pragmatic Interventions to Reduce Alarm Frequency: Review of Physiologic Monitor Alarms. J. Hosp. Med..

[3] Azimi I., Pahikkala T., Rahmani A.M., Niela-Vilén H., Axelin A., Liljeberg P. (2019). Missing data resilient decision-making for healthcare IoT through personalization: A case study on maternal health. Future Gener. Comput. Syst..

[4] Kumar R.B., Goren N.D., Stark D.E., Wall D.P., Longhurst C.A. (2016). Automated integration of continuous glucose monitor data in the electronic health record using consumer technology. J. Am. Med Inform. Assoc..

[5] Moody G.B. The PhysioNet/Computing in Cardiology Challenge 2010: Mind the Gap. Proceedings of the Computing in Cardiology.

[6] Shivers J.P., Mackowiak L., Anhalt H., Zisser H. (2013). “Turn it Off!”: Diabetes Device Alarm Fatigue Considerations for the Present and the Future. J. Diabetes Sci. Technol..

[7] Keller J.P. (2012). Clinical alarm hazards: A “top ten” health technology safety concern. J. Electrocardiol..

[8] Johnson K.R., Hagadorn J.I., Sink D.W. (2017). Alarm Safety and Alarm Fatigue. Clin. Perinatol..

[9] Shannon C.E. (1948). A Mathematical Theory of Communication. Bell Syst. Tech. J..

[10] Pincus S.M. (1991). Approximate entropy as a measure of system complexity. Proc. Natl. Acad. Sci. USA.

[11] Richman J.S., Moorman J.R. (2000). Physiological time-series analysis using approximate entropy and sample entropy. Am. J. Physiol.-Heart Circ. Physiol..

[12] Bandt C., Pompe B. (2002). Permutation Entropy: A Natural Complexity Measure for Time Series. Phys. Rev. Lett..

[13] Chen W., Wang Z., Xie H., Yu W. (2007). Characterization of Surface EMG Signal Based on Fuzzy Entropy. IEEE Trans. Neural Syst. Rehabil. Eng..

[14] Rostaghi M., Azami H. (2016). Dispersion Entropy: A Measure for Time-Series Analysis. IEEE Signal Process. Lett..

[15] Caldirola D., Bellodi L., Caumo A., Migliarese G., Perna G. (2004). Approximate Entropy of Respiratory Patterns in Panic Disorder. Am. J. Psychiatry.

[16] Lake D.E., Richman J.S., Griffin M.P., Moorman J.R. (2002). Sample entropy analysis of neonatal heart rate variability. Am. J. Physiol.-Regul. Integr. Comp. Physiol..

[17] Olofsen E., Sleigh J., Dahan A. (2008). Permutation entropy of the electroencephalogram: A measure of anaesthetic drug effect. Br. J. Anaesth..

[18] Cirugeda-Roldan E., Cuesta-Frau D., Miro-Martinez P., Oltra-Crespo S. (2014). Comparative Study of Entropy Sensitivity to Missing Biosignal Data. Entropy.

[19] Dong X., Chen C., Geng Q., Cao Z., Chen X., Lin J., Jin Y., Zhang Z., Shi Y., Zhang X.D. (2019). An Improved Method of Handling Missing Values in the Analysis of Sample Entropy for Continuous Monitoring of Physiological Signals. Entropy.

[20] Garcia-Gonzalez M., Fernandez-Chimeno M., Ramos-Castro J. (2009). Errors in the Estimation of Approximate Entropy and Other Recurrence-Plot-Derived Indices Due to the Finite Resolution of RR Time Series. IEEE Trans. Biomed. Eng..

[21] Molina-Picó A., Cuesta-Frau D., Aboy M., Crespo C., Miró-Martínez P., Oltra-Crespo S. (2011). Comparative study of approximate entropy and sample entropy robustness to spikes. Artif. Intell. Med..

[22] Azami H., Escudero J. (2018). Amplitude- and Fluctuation-Based Dispersion Entropy. Entropy.

[23] Rostaghi M., Ashory M.R., Azami H. (2019). Application of dispersion entropy to status characterization of rotary machines. J. Sound Vib..

[24] Kim K.K., Baek H.J., Lim Y.G., Park K.S. (2012). Effect of missing RR-interval data on nonlinear heart rate variability analysis. Comput. Methods Programs Biomed..

[25] Rousseeuw P.J., Croux C. (1993). Alternatives to the Median Absolute Deviation. J. Am. Stat. Assoc..

[26] Leys C., Ley C., Klein O., Bernard P., Licata L. (2013). Detecting outliers: Do not use standard deviation around the mean, use absolute deviation around the median. J. Exp. Soc. Psychol..

[27] Pontet J., Contreras P., Curbelo A., Medina J., Noveri S., Bentancourt S., Migliaro E.R. (2003). Heart rate variability as early marker of multiple organ dysfunction syndrome in septic patients. J. Crit. Care.

[28] Augustyniak P. (2011). Wearable wireless heart rate monitor for continuous long-term variability studies. J. Electrocardiol..

[29] Iyengar N., Peng C.K., Morin R., Goldberger A.L., Lipsitz L.A. (1996). Age-related alterations in the fractal scaling of cardiac interbeat interval dynamics. Am. J. Physiol.-Regul. Integr. Comp. Physiol..

[30] Goldberger A.L., Amaral L.A.N., Glass L., Hausdorff J.M., Ivanov P.C., Mark R.G., Mietus J.E., Moody G.B., Peng C.K., Stanley H.E. (2000). PhysioBank, PhysioToolkit, and PhysioNet: Components of a New Research Resource for Complex Physiologic Signals. Circulation.

[31] Shoeb A.H. (2009). Application of Machine Learning to Epileptic Seizure Onset Detection and Treatment. Ph.D. Thesis.

[32] Pimentel M.A.F., Johnson A.E.W., Charlton P.H., Birrenkott D., Watkinson P.J., Tarassenko L., Clifton D.A. (2017). Toward a Robust Estimation of Respiratory Rate From Pulse Oximeters. IEEE Trans. Biomed. Eng..

[33] Kafantaris E., Piper I., Lo T.Y.M., Escudero J. (2019). Application of Dispersion Entropy to Healthy and Pathological Heartbeat ECG Segments. Proceedings of the 2019 41st Annual International Conference of the IEEE Engineering in Medicine and Biology Society (EMBC).

[34] Rajendra Acharya U., Paul Joseph K., Kannathal N., Lim C.M., Suri J.S. (2006). Heart rate variability: A review. Med. Biol. Eng. Comput..

[35] Kaczka D.W., Barnas G.M., Suki B., Lutchen K.R. (1995). Assessment of time-domain analyses for estimation of low-frequency respiratory mechanical properties and impedance spectra. Ann. Biomed. Eng..

[36] Diong B., Nazeran H., Nava P., Goldman M. (2007). Modeling Human Respiratory Impedance. IEEE Eng. Med. Biol. Mag..

[37] Dressler O., Schneider G., Stockmanns G., Kochs E. (2004). Awareness and the EEG power spectrum: Analysis of frequencies. Br. J. Anaesth..

